# Advances in the clinical application of bispecific antibodies in cancer therapy

**DOI:** 10.1016/j.isci.2025.114203

**Published:** 2025-11-25

**Authors:** Shiqi Zhou, Feng Li, Mengke Niu, Kongming Wu, Tianye Li, Ming Yi

**Affiliations:** 1 Department of Gynecology, The Second Affiliated Hospital, Zhejiang University School of Medicine, Zhejiang University, Hangzhou, China; 2 Department of Liver Surgery and Transplantation, Liver Cancer Institute, Zhongshan Hospital, Fudan University, Key Laboratory of Carcinogenesis and Cancer Invasion, Ministry of Education, Shanghai, China; 3 Department of Pulmonary Surgery, Zhejiang Cancer Hospital, Hangzhou Institute of Medicine (HIM), Chinese Academy of Sciences, Hangzhou, China; 4 Department of Breast Surgery, The First Affiliated Hospital, College of Medicine, Zhejiang University, Hangzhou, China; 5 Cancer Center, Shanxi Bethune Hospital, Shanxi Academy of Medical Science, Tongji Shanxi Hospital, Third Hospital of Shanxi Medical University, Shanxi, China

**Keywords:** Oncology, Therapeutics, Cellular therapy

## Abstract

Cancer immunotherapy has emerged as one of the most groundbreaking advancements. However, the tumor microenvironment (TME) is often dominated by various immunosuppressive factors, compromising the efficacy of single-target therapies, leading to non-responsiveness or resistance. Bispecific antibodies (BsAbs) represent an innovative immunotherapeutic strategy with enormous potential for improving cancer treatment outcomes. Unlike monoclonal antibodies, BsAbs can concurrently inhibit multiple pro-tumor pathways, target immune checkpoints to mitigate resistance, and bind to two distinct antigens, thereby enhancing specificity while minimizing off-target effects. Moreover, BsAbs are more cost-efficient and less toxic compared to the use of two separate monoclonal antibodies in combination. In recent decades, BsAbs have made remarkable progress in clinical development. Several BsAbs, such as blinatumomab, mosunetuzumab, teclistamab, glofitamab, epcoritamab, talquetamab, ivonescimab, cadonilimab, tarlatamab, zenocutuzumab, and catumaxomab, have achieved notable success in clinical trials. This review highlights clinically approved BsAbs and provides a comprehensive summary of their therapeutic applications in cancer treatment.

## Graphical abstract

**Figure:**
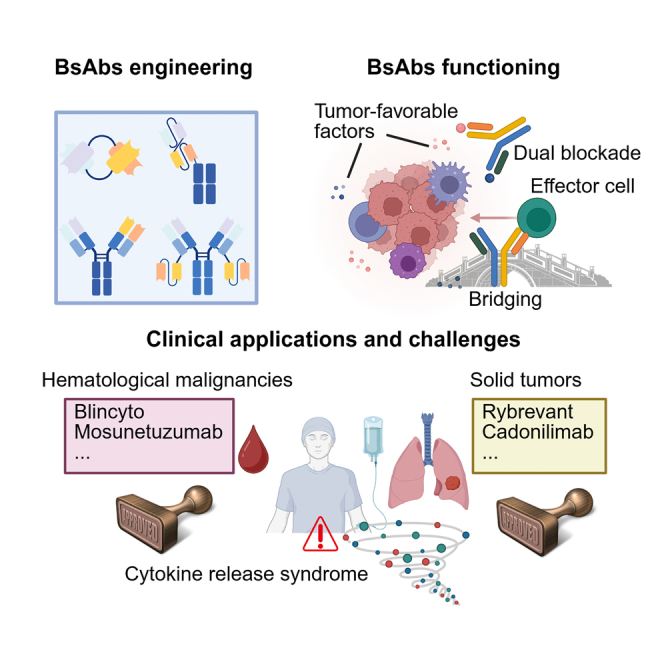

## Introduction

Cancer remains a leading cause of global mortality.^1^ Traditional modalities (surgery, radiotherapy, and chemotherapy) have improved outcomes, but advanced and recurrent cancers remain difficult to cure due to tumor heterogeneity and complex molecular alterations.^2^ For refractory disease, immunotherapies—including monoclonal antibodies, cancer vaccines, oncolytic viruses, chimeric antibody receptor cell therapies, antibody-drug conjugates (ADCs), and bispecific antibodies (BsAbs)—offer complementary mechanisms that can overcome limitations of single-target approaches.^3^^,^^4^^,^^5^^,^^6^^,^^7^^,^^8^ Compared to monoclonal antibodies, BsAbs offer several advantages: they simultaneously block multiple pro-tumor pathways, enhancing therapeutic efficacy and relieving resistance; dual targeting of immune checkpoints helps prevent immune escape; binding to two different antigens on cancer cells improves targeting specificity and minimizes off-target effects; they redirect T cells to enhance antitumor responses; and they are more cost-effective and less toxic than combining two separate monoclonal antibodies.^9^^,^^10^ This review focuses on BsAbs, positioning them as the next-generation strategy in antibody-based cancer immunotherapy.

BsAbs are engineered from two or more parent antibodies, enabling them to simultaneously target two distinct antigens. Unlike natural antibodies, which are bivalent and monospecific—binding to a single specific antigen—BsAbs possess the unique ability to engage two different targets concurrently.^11^ Additionally, BsAbs possess the ability to direct immune effector cells to target cancer cells with high specificity. They achieve this by simultaneously binding one antigen on the cancer cell and another on immune cells, such as natural killer (NK) cells or effector T cells.^12^^,^^13^^,^^14^

In recent years, BsAbs have made significant strides, establishing themselves as dynamic and highly adaptable therapeutic agents with extensive applications across various medical fields.^15^^,^^16^ A burgeoning pipeline of BsAbs is advancing through multiple phases of clinical trials, underscoring their versatile design and ability to tackle complex, multifactorial disease mechanisms.^17^ Despite clinical success in hematologic malignancies, translating BsAbs to solid tumors remains challenging because of the immunosuppressive tumor microenvironment (TME).^18^^,^^19^ The TME is marked by a dense network of immunosuppressive cells, regulatory molecules, and abnormal vasculature, which collectively hinder T cell infiltration and function.^20^^,^^21^ These adverse conditions not only diminish the clinical efficacy of BsAbs but also reduce the overall responsiveness of tumors to immunotherapeutic interventions.^22^^,^^23^

In this context, this review will explore the landscape of approved BsAbs. By examining their structural diversity and clinical applications, we aim to provide insights into how BsAbs are transforming cancer therapy and to highlight their evolving role within the broader immuno-oncology framework.

## BsAb format

**Figure 1:**
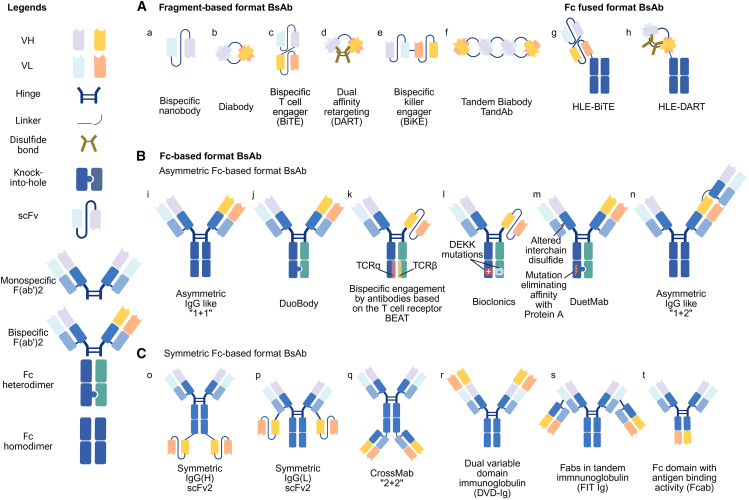
Landscape of BsAb formats and design elements (A) These subtypes of BsAbs are mainly constituted with Fab/scFv but without Fc. Fc fused fromat falls in between fragment-based and Fc-based format with Fab/scFv grafting on the Fc skeleton. (a–f) (Left) Legend of building blocks: VH, VL, hinge, linker, disulfide bond, knobs-into-holes (KIH) for Fc heterodimerization, and single-chain variable fragment (scFv). (Top) Fragment-based BsAb formats without Fc: bispecific nanobody (a), diabody (b), BiTE (c), DART (d), bispecific killer engager (BiKE) (e), and TandAb (f). (g–n) (Upper right) Fc-fused BsAb: half-life-extended (HLE) BiTE (g) and HLE-DART (h) created by fusing fragments to an Fc for FcRn-mediated recycling. (B) These subtypes of BsAbs retain intact IgG framework and Fc region, which further devide into asymmetric and symmertric format (C) based on the symmertry of antibody construction. (Middle) Asymmetric Fc-based BsAb: IgG-like 1 + 1 (i), DuoBody (j), BEAT using TCRα/β constant-domain pairing (k), and Bioclonics with DEKK electrostatic mutations (l). Formats with altered inter-chain disulfide, variants carrying Protein-A-binding ablating mutations for selective purification: DuetMab (m) and IgG-like 1 + 2 (n). (o–t) (Bottom) Symmetric Fc-based BsAb: IgG(H)-scFv_2_ (o) and IgG(L)-scFv_2_ (scFv fused to the heavy- or light-chain C terminus) (p), CrossMab 2 + 2 (q), DVD-Ig (r), FIT-Ig (s), and Fc domain with antigen-binding activity (Fcab) (t). Formats differ in valency and geometry—1+1 (monovalent for each specificity), 1 + 2 (bivalent for one specificity), 2 + 2 (bivalent for both)—as well as in chain-pairing strategies. These architectures enable functions ranging from pure ligand/receptor blockade to potent cell-bridging and receptor clustering while allowing tuning of pharmacokinetics and effector functions (created with BioRender.com).

Antibodies are divided into two main regions: the fragment crystallizable (Fc) region,^24^ which interacts with Fc receptors to initiate immune responses, and the fragment antigen-binding (Fab) region, which contains variable fragments responsible for antigen binding.^25^ Jointly, the heavy and light chains’ variable regions create what are known as single-chain variable fragments (scFvs). The development of BsAbs, which have two separate binding sites, has opened up new possibilities in the fight against cancer.^11^ Recent technological advancements have enabled the development of various BsAb formats,^26^ which can generally be divided into fragment-based (Figure 1A) and Fc-based categories, with the latter further subdivided into asymmetric (Figure 1B) and symmetric Fc-based formats (Figure 1C).

### Fragment-based BsAbs

Fragment-based BsAbs provide a streamlined approach to generating bispecific molecules by incorporating different antigen-binding components, like fragments of antibodies, into one cohesive construct. By lacking the Fc domain, they avoid the chain-pairing issues seen in full-length antibodies, simplifying production in eukaryotic or bacterial systems^13^ (Figure 1A). This modular design reduces manufacturing cost, enables flexible valency, and allows rapid customization for diverse therapeutic purposes. However, the absence of an Fc region precludes FcRn-mediated recycling, resulting in a shorter serum half-life.^27^

A bispecific nanobody comprises two distinct nanobodies fused into one single-chain construct, each conferring unique binding specificity (Figure 1Aa). Similarly, a diabody is a compact antibody fragment engineered for bivalent or bispecific binding (Figure 1Ab). These formats belong to a broader category of fragment-based BsAbs that also includes bispecific T cell engagers (BiTEs), tandem diabodies (TandAbs), and dual-affinity retargeting (DART) molecules.^28^ The two scFvs in a BiTE are connected in a way that allows one to attach to the CD3 on T cells and the other to a tumor-associated antigen (TAA), which allows the T cells to directly attack the tumor cells (Figure 1Ac).^8^^,^^29^^,^^30^ DARTs (Figure 1Ad) have a diabody backbone carrying two polypeptide chains, with the VH connected by a disulfide bond, leading to a more stable molecule with potentially higher levels of T cell activation.^31^^,^^32^ Similarly to BiTEs, BiKEs are heterodimeric bispecific scFvs that target NK cells (Figure 1Ae). TandAbs (Figure 1Af) incorporate two diabodies within a single molecule, enabling the simultaneous engagement of two antigens and promoting potent signaling.^33^^,^^34^^,^^35^ Emerging half-life extension (HLE) strategies counter the rapid clearance of fragment-based BsAbs. In one approach, the HLE-BiTE AMG673 (CD3×CD33) gains a ∼21-day half-life by fusing an IgG Fc region to its N terminus. Fc integration is also used in second-generation HLE-BiTE molecules to prolong exposure, now in preclinical/clinical trials (NCT05740566)^36^ (Figure 1Ag). The HLE-DART format follows the same principle, fusing the core structure to an Fc domain or other moieties to boost serum persistence (Figure 1Ah).

### Fc-based BsAbs

Fc-based BsAbs incorporate the Fc region of immunoglobulins, conferring advantages such as prolonged serum half-life, improved stability, and effector functions^37^ Through diverse Fc-engineering strategies, these molecules acquire optimized biological and physicochemical properties.^38^ Broadly, Fc-based BsAbs can be divided into asymmetric and symmetric formats (Figures 1B and 1C).

Asymmetric Fc-based BsAbs require specialized engineering to ensure correct heterodimer formation in the Fc region. This is commonly achieved through CH3 domain modifications or the use of common light chains to prevent chain mispairing.^15^^,^^39^ Representative asymmetric architectures include asymmetric IgG-like (1 + 1) and (1 + 2) formats (Figures 1Bi and 1Bn); DuoBody, which relies on controlled Fab-arm exchange (Figure 1Bj); BEAT (bispecific engagement by antibodies based on the T cell receptor [TCR]), which mimics TCR heterodimerization (Figure 1Bk); Bioclonics, using DEKK mutations to promote heterodimerization (Figure 1Bl); and DuetMab, incorporating disulfide and affinity engineering to stabilize the Fc heterodimers (Figure 1Bm).

Several key engineering strategies underlie these designs. The “knobs-into-holes” (KiH) approach, a widely used CH3 engineering technique, facilitates correct Fc heterodimer assembly by introducing a protruding “knob” mutation in one heavy chain and a complementary “hole” mutation in the other.^40^ The common light chain (LC) strategy simplifies the molecular structure by allowing a single LC to pair with both heavy chains, thereby enhancing stability and minimizing mispairing.^41^ The CrossMab approach addresses LC mispairing by swapping CH1 and CL domains in one Fab arm, creating distinct Fab regions and ensuring correct pairing^42^

Symmetric Fc-based BsAbs, in contrast, closely resemble native antibodies, with additional antigen-binding modules (e.g., scFv or Fv domains) grafted onto the N or C termini of the IgG framework.^43^^,^^44^^,^^45^ Representative symmetric formats include symmetric IgG(H)-scFv2 and IgG(L)-scFv2, with scFv fused to heavy or light chains (Figures 1Co and 1Cp); CrossMab (2 + 2), which enables correct heavy-light chain pairing (Figure 1Cq); dual variable domain immunoglobulin (DVD-Ig), incorporating tandem variable domains (Figure 1Cr); Fabs in tandem immunoglobulin (FIT-Ig), combining multiple Fab modules (Figure 1Cs); and Fc domain with antigen-binding activity (Fcab), where the Fc domain itself contributes to target recognition (Figure 1Ct). The biophysical properties of these constructs depend on the characteristics and attachment sites of appended fragments.^46^

## Functional subtypes of BsAbs based on mechanism of action

In addition to structural formats, BsAbs can also be classified according to their primary mechanism of action. This functional categorization reflects their immunological targets, mode of immune engagement, and potential therapeutic indications. The major functional subtypes are as follows.

### CD3-engaging BsAbs (T cell engagers)

These BsAbs simultaneously bind a TAA and CD3ε on T cells, bridging cytotoxic lymphocytes to tumor cells and inducing major histocompatibility complex (MHC)-independent killing. This mechanism leads to potent T cell activation and tumor lysis. Blinatumomab (CD19×CD3) and glofitamab (CD20×CD3) are approved examples demonstrating significant clinical benefit in B cell malignancies.^47^^,^^48^

### Dual immune checkpoint-blocking BsAbs

These molecules simultaneously block two inhibitory pathways, such as PD-1/CTLA-4, to restore T cell activation and enhance immune infiltration. Cadonilimab (PD-1×CTLA-4) has shown encouraging activity across multiple tumor types.^49^^,^^50^^,^^51^ Preclinical studies suggest that combining checkpoint blockade in a single molecule may improve pharmacokinetics and reduce immune-related toxicity compared with dual-antibody regimens.^52^

### Co-stimulatory BsAbs

These antibodies provide dual signals—TAA recognition (“signal 1”) and co-stimulatory receptor engagement (“signal 2”)—to potentiate T cell proliferation and survival. Prominent examples include 4-1BB×TAA and CD28×TAA BsAbs under early clinical evaluation, often combined with PD-1 blockade.^53^^,^^54^

### NK cell-engaging BsAbs

By engaging activating NK cell receptors (e.g., CD16A/FcγRIIIa) and TAAs, these BsAbs trigger antibody-dependent cellular cytotoxicity (ADCC) and cytokine secretion. They complement T-cell-based immunity and may benefit tumors with low MHC-I expression.^55^^,^^56^

### Tumor-stroma dual-targeting BsAbs

These BsAbs target both tumor antigens and stromal or vascular components such as fibroblast activation protein (FAP) or vascular endothelial growth factor (VEGF), aiming to remodel the TME. By disrupting stromal barriers and normalizing vasculature, they enhance immune cell infiltration and antibody penetration, resulting in stronger antitumor activity in preclinical models.^57^^,^^58^

### Dual tumor antigen-targeting BsAbs

By recognizing two TAAs on the same tumor cell, these BsAbs enhance binding avidity and reduce antigen-loss escape. Amivantamab (EGFR×MET) exemplifies this approach, overcoming oncogenic signaling and resistance in EGFR-mutant non-small cell lung cancer (NSCLC).^59^ Other designs, such as HER2×HER3 BsAbs, inhibit receptor dimerization and downstream signaling.^60^

### BsAbs incorporating immune modulators or cytokine payloads

These next-generation BsAbs integrate cytokine payloads (e.g., IL-15 superagonists or engineered IL-2 variants) to locally boost immune activation within the TME, maximizing efficacy while minimizing systemic toxicity. Early studies of IL-15×CD20 and IL-2 variant×PD-1 BsAbs demonstrate potent immune activation and tumor regression in preclinical models.^61^^,^^62^

## Antigen selection strategies for bispecific antibodies

The therapeutic efficacy and safety of BsAbs largely depend on the rational selection of target antigens on tumor and immune cells. Understanding the principles of antigen selection is essential for optimizing BsAb design and clinical translation.

### Tumor-associated antigens

Optimal TAAs should exhibit high tumor selectivity and minimal expression in normal tissues to reduce on-target/off-tumor toxicity. High surface antigen density facilitates stable immune synapse formation and enhances cytotoxic efficacy.^63^ The internalization kinetics of target antigens also affects BsAb performance: rapidly internalizing antigens (e.g., HER2) may limit surface engagement but can be exploited for payload delivery formats such as ADC-like BsAbs.^64^ Tumor heterogeneity represents a major barrier, as antigen loss variants can drive resistance. Dual-antigen targeting approaches improve tumor coverage and mitigate immune escape in heterogeneous models.^65^ Emerging targets, including Trop-2, B7-H3, ICAM-1, and GPRC5D, show restricted normal tissue expression and broad tumor prevalence, making them promising candidates for next-generation BsAbs.^66^^,^^67^^,^^68^

### Immune cell targets

On the immune effector side, CD3 remains the most validated target for T-cell-engaging BsAbs. However, alternative receptors are under active exploration to fine-tune T cell activation and mitigate cytokine release syndrome (CRS).^19^^,^^69^ To enhance selectivity and safety, co-stimulatory receptors such as CD28, 4-1BB, and ICOS are being incorporated into BsAb designs, enabling the delivery of conditional secondary signals within the TME.^70^ Beyond T cells, NK-cell receptors (e.g., CD16A, NKp46, and NKG2D) are being leveraged to mediate ADCC and broaden immune effector engagement.^71^ In parallel, BsAbs targeting inhibitory checkpoints (e.g., PD-1, PD-L1, CTLA-4, LAG-3, and TIGIT) are being developed to reinvigorate exhausted immune cells through combined tumor-specific engagement.^72^

### Design considerations

Antigen selection for BsAbs should follow the following key principles: (1) tumor specificity and safety margin—limit expression in healthy tissues, (2) antigen density and stability—ensure robust immune synapse formation, (3) internalization kinetics—balance surface retention vs. suitability for payload delivery, (4) tumor heterogeneity and escape mechanisms—address antigen loss via dual targeting, and (5) effector pathway compatibility—align tumor targets with immune cell receptors to maximize synergy (Table 1).

**Table 1:** Representative antigens for BsAb development and their selection rationale

Category	Target antigen(s)	Rationale/Selection criteria	Clinical development
T cell engaging BsAbs	CD3	Pan-T cell engagement; robust cytotoxic synapse formation	Validated in blinatumomab,^73^ teclistamab^74^
Co-stimulatory targets	CD28, 4-1BB, ICOS	Provide conditional secondary signals to enhance T cell activation	EGFR×CD28 (REGN7075)^54^; PD-L1×CD28 (NI-3201)^75^
NK cell engaging targets	CD16A, NKp46, NKG2D	Promote ADCC and innate cytotoxicity; broaden effector repertoire	EGFR×CD16A (AFM24)^56^
Classical tumor antigens	CD20, BCMA, EGFR, MET	High density; lineage restricted or tumor enriched; validated in hematologic malignancies & solid tumors	Multiple approved (e.g., CD20×CD3,^76^ BCMA×CD3,^77^ EGFR×MET BsAbs^59^)
Dual-target solid tumor antigens	HER2/HER3, EGFR/MET	Overcome resistance and tumor heterogeneity; limit antigen escape	HER2/HER3 BsAbs^60^; EGFR/MET BsAbs^59^
Emerging solid tumor antigens	Trop-2, B7-H3, ICAM-1, GPRC5D	Broad expression in solid tumors; limited in vital tissues; high translational potential	Trop-2×CD3^66^; B7-H3 BsAbs^67^; GPRC5D^68^
Checkpoint modulators	PD-1, PD-L1, CTLA-4, LAG-3, TIGIT	Release inhibitory brakes on T cells; synergize with tumor-directed arms	Multiple BsAbs (e.g., PD-1×CTLA-4^49^)
Stromal/angiogenic targets	VEGF, FAP	Normalize tumor vasculature; reduce stromal immunosuppression	VEGF×PD-1 BsAbs^78^

## Mechanisms of action of bispecific antibodies

BsAbs deploy multiple, interlocking immunological mechanisms to convert the TME from an immune-excluded or suppressed state into one permissive for cytotoxic elimination. Below, we summarize key cellular interactions and signaling events that underlie clinical activity across BsAb classes.

### T cell redirection and immune-synapse formation

CD3-engaging BsAbs physically bridge cytotoxic T cells and tumor cells by binding CD3ε and a TAA, forming a functional immune synapse characterized by TCR microcluster assembly, LFA-1-ICAM-1 adhesion, and directed release of perforin and granzymes that induce tumor cell apoptosis. This interaction occurs independently of MHC restriction and bypasses co-stimulatory requirements, explaining the rapid cytotoxic responses observed with T-cell-redirecting BsAbs.^14^^,^^79^

### Cytokine release and immune amplification

Following BsAb engagement, activated T cells release cytokines including interferon (IFN)-γ, tumor necrosis factor (TNF)-α, and interleukin (IL)-2, which promote effector proliferation, upregulate adhesion molecules, and recruit additional immune subsets. This cytokine milieu not only enhances antitumor immunity but also contributes to systemic toxicities such as CRS.^69^^,^^80^

### Recruitment of accessory effector cells

Beyond T cells, BsAbs can recruit NK cells (CD16A×TAA) and macrophages (via Fcγ receptors), expanding the effector network through ADCC and phagocytosis.^81^ For example, NK cell-engaging BsAbs trigger degranulation and IFN-γ secretion, while macrophage activation enhances antigen presentation and supports cross-priming of adaptive responses.^82^

### Co-stimulatory signaling and checkpoint modulation

Certain BsAbs deliver conditional co-stimulatory signals (e.g., CD28×TAA) that augment T cell activation only upon tumor recognition, minimizing off-tumor toxicity. Others block inhibitory immune checkpoints (PD-1/PD-L1, CTLA-4) to restore effector function. These dual-targeting strategies synergize with cytotoxic mechanisms to enhance the magnitude and durability of antitumor immunity.^72^

### Remodeling stromal and angiogenic barriers

Tumor-stroma dual-targeting BsAbs simultaneously engage TAAs and stromal or angiogenic components within the TME (e.g., FAP, VEGF). By disrupting stromal-tumor crosstalk, these BsAbs can normalize abnormal vasculature, enhance immune cell infiltration, and relieve stromal-mediated immune suppression, thereby overcoming physical and biochemical barriers that limit immunotherapy efficacy.^57^^,^^83^

### Illustrative schematic

**Figure 2:**
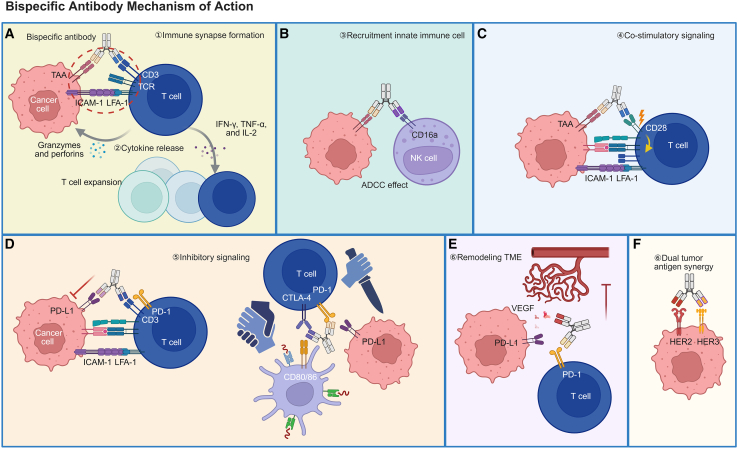
Mechanisms of action of BsAbs in cancer immunotherapy. BsAbs exert antitumor effects through multiple coordinated mechanisms (A) Immune synapse formation (①): BsAbs bridge tumor-associated antigens (TAAs) on cancer cells with CD3 on T cells, promoting immune synapse assembly, T cell activation, cytokine release (IFN-γ, TNF-α, and IL-2), and cytotoxic granzyme/perforin secretion (②). (B) Recruitment of innate immune cells: by engaging CD16a on NK cells, BsAbs can trigger ADCC. (C) Co-stimulatory signaling: BsAbs targeting TAA and co-stimulatory receptors such as CD28 enhance T cell activation independently of MHC presentation. (D) Inhibitory signaling blockade: BsAbs simultaneously block inhibitory checkpoints such as PD-1/PD-L1 or CTLA-4, restoring T cell effector function. (E) Remodeling of the TME: BsAbs that neutralize VEGF normalize tumor vasculature and enhance immune infiltration. (F) Dual tumor antigen synergy: BsAbs co-target distinct TAAs (e.g., HER2 and HER3), improving tumor specificity and minimizing antigen escape. (Created with BioRender.com).

A schematic diagram is recommended here, depicting the stepwise process: (1) BsAb-mediated bridging of T cells and tumor cells, (2) immune synapse formation, (3) perforin/granzyme release, (4) cytokine secretion, (5) recruitment of NK cells and macrophages, and (6) co-stimulatory and checkpoint-modulating pathways. This visual aid would enhance comprehension of the multifaceted immune mechanisms underlying BsAb function (Figure 2).

## Clinical applications in cancer

BsAbs can simultaneously bind two different antigens or epitopes on the same antigen. By structurally bridging target cells with immune cells, they enable precise immune targeting and dual blockade of disease-related signaling pathways.^13^^,^^46^^,^^84^^,^^85^ This dual-targeting approach enhances therapeutic potency by recruiting immune cells to tumors, disrupting oncogenic signals, or directly inducing cytotoxicity.^86^ Over the past decade, BsAbs have gained considerable attention in oncology, leading to significant mechanistic and technological advances. As of the end of 2024, 17 BsAbs have been approved globally, 14 of which are used in cancer therapy (Table 2), highlighting their growing role in expanding treatment options for patients with resistant or hard-to-treat tumors.

**Table 2:** Bispecific antibodies in clinical trials for cancer (data obtained from ClinicalTrials.gov)

Drugs	Sponsor	Phase	Code		Diseases	Number of patients	Clinical outcomes	Approval (region/year)
Blinatumomab	Amgen	2	NCT01209286	Completed	ALL	36	CR: 69%; median RFS: 7.6 m; median OS: 9.8 m	FDA: 2014; EMA:2015; NMPA: 2020
		2	NCT01466179	Completed	ALL	189	CR: 43%; median RFS: 5.9 m; median OS: 6.1 m	
		1/2	NCT01471782	Completed	ALL	70	CR: 39%; median RFS: 4.4 m; median OS: 7.5 m	
		3	NCT02013167	Terminated	ALL	405	CR: 34%; median RFS: 7.3 m; median OS: 7.7 m	
		2	NCT01207388	Completed	ALL with MRD	116	CR: 78%; median RFS: 18.9 m; median OS: 36.5 m	
		2	NCT02744768	Unknown	ALL (combined with dasatinib)	63	CR: 98%; median RFS: NR; median OS: NR	
		3	NCT02101853	Active, not recruiting	ALL	669	CR: 80.3%; median RFS: NR; median OS: NR	
		3	NCT02393859	Completed	ALL	108	CR: 88.9%; median RFS: NR; median OS: NR	
		2	NCT03263572	Recruiting	ALL	60	CR: 68.3%; median RFS: NR; median OS: NR	
		3	NCT02003222	Active, not recruiting	MRD-negative ALL	224	CR: NA; median RFS: NR; median OS: NR	
		3	NCT01741792	Completed	DLBCL	25	CR: 19%; median RFS: 3.7 m; median OS: 5.0 m	
		2	NCT02000427	Completed	ALL	45	CR: 36%; median RFS: 6.7 m; median OS: 7.1 m	
Mosunetuzumab	Roche	1/2	NCT02500407	Active, not recruiting	B-NHLs	197	Aggressive B-NHL: CR: 19.4%; median RFS: 1.4 m; median OS: NR; indolent B-NHL: CR: 48.5%; median RFS: 11.8 m; median OS: NR	FDA: 2022; EMA:2022; NMPA: NA
		2	NCT03677141	Completed	DLBCL	40	CR: 90%; median RFS: NR; median OS: NR	
		1b/2	NCT03671018	Active, not recruiting	LBCL	120	CR: 45.9%; median RFS: 11.4 m; median OS: 23.3 m	
Teclistamab	Janssen	1	NCT03145181	Active, not recruiting	MM	40	CR: 40%; median RFS: NA; median OS: NA	FDA: 2022; EMA:2022; NMPA: 2024
		2	NCT04557098	Active, not recruiting	MM	165	CR: 39.4%; median RFS: 11.3 m; median OS: 18.3 m	
Glofitamab	Roche	1/2	NCT03075696	Recruiting	DLBCL	155	CR: 37%; median RFS: 3.8 m; median OS: 11.5 m	FDA: 2023; EMA:2023; NMPA: 2023
		3	NCT04408638	Active, not recruiting	DLBCL	274	CR: 58.5%; median RFS: 13.8 m; median OS: 25.5 m	
Epcoritamab	Genmab	1/2	NCT03625037	Active, not recruiting	LBCL	157	CR: 38.9%; median RFS: 4.4 m; median OS: NR	FDA: 2023; EMA:2023; NMPA: NA
Talquetamab	Janssen	1/2	NCT03399799/NCT04634552	Active, not recruiting	MM	375	0.4 mg/kg: CR: 33%, median RFS: 7.5 m, median OS: NA; 0.8 mg/kg: CR: 40%, median RFS: 11.2 m, median OS: NA; with previous TCR: CR: 41%, median RFS: 7.7 m; median OS: NA	FDA: 2023; EMA:2023; NMPA: NA
Elranatamab	Pfizer	2	NCT04649359	Active, not recruiting	MM	123	CR: 35.0%; median RFS: NA; median OS: NA	FDA: 2023; EMA:2023; NMPA: NA
		1	NCT03269136	Completed	MM	55	CR: 38.2%; median RFS: 11.8 m; median OS: 21.2 m	
Amivantamab	Janssen	1	NCT02609776	Active, not recruiting	NSCLC	81	CR: 4%; median RFS: 8.3 m; median OS: 22.8 m	FDA: 2021; EMA:2021; NMPA: 2025
		3	NCT04538664	Active, not recruiting	NSCLC	153	CR: 4%; median RFS: 11.4 m; median OS: NA	
		3	NCT05388669	Active, not recruiting	NSCLC	418	Subcutaneous group: CR: 0.5%, median RFS: 6.1 m, median OS: 12.9; intravenous group: CR: 0.5%, median RFS: 4.3 m, median OS: NA	
		3	NCT04487080	Active, not recruiting	NSCLC	429	CR: 7%; median RFS: 23.7 m, median OS: NA	
Tebentafusp	Immunocore	3	NCT03070392	Active, not recruiting	Uveal melanoma	252	CR: 0.4%; median RFS: 3.3 m, median OS: 21.7 m	FDA: 2022; EMA:2022; NMPA: NA
		2	NCT02570308	Completed	Uveal melanoma	127	CR: NR; median RFS: 2.76 m; median OS: 16.8 m	
		1b	NCT02535078	Withdrawn	Uveal melanoma	85	CR: 1%; median RFS: NR; median OS: 18.7	
Ivonescimab	Akeso Biopharma	1b	NCT04900363	Active, not recruiting	NSCLC	108	CR: NR; median RFS: 11.4; median OS: NR	FDA: NA; EMA: NA; NMPA: 2024
		1a	NCT04047290	Completed	NSCLC	51	CR: NR; median RFS: NR; median OS: NR	
		3	NCT05184712	Active, not recruiting	NSCLC	161	CR: NR; median RFS: 7.1 m; median OS: NR	
Cadonilimab	Akeso Biopharma	1b/2	NCT04172454	Completed	NSCLC	53	CR: NR; median RFS: 1.9 m; median OS: 13.3 m	FDA: NA; EMA: NA; NMPA: 2022
		1b/2	NCT03852251	Completed	GC, GEJC	338	CR: NR; median RFS: NR; median OS: NR	
		1	NCT03261011	Completed	Solid tumors	119	CR: 1.7%; median RFS: NR; median OS: NR	
		1b/2	NCT04444167	Completed	HCC	59	CR: 0%; median RFS: 9.7 m; median OS: 26.9 m	
		1b/2	NCT04646330	Completed	NSCLC	69	CR: 0%; median RFS: NA; median OS: NR	
		2	NCT04868708	Completed	CC	45	Cohort A-15: CR: 20%, median RFS: 11.1 m, median OS: NR; cohort A-10: CR: 6.25%, median RFS: 7.06 m, median OS: NR; cohort B-10: CR: 7.7%, median RFS: NR; median OS: NR	
		1b/2	CTR20182027	/	GC, GEJC	98	CR: 4.3%; median RFS: 8.18 m; median OS: 17.48 m	
		3	NCT04982237	Active, not recruiting	CC	222	CR: 36%; median RFS: 12.7 m; median OS: NR	
Tarlatamab	Amgen	2	NCT03319940	Active, not recruiting	SCLC	107	CR: 2%; median RFS: 3.7 m; median OS: 13.2 m	FDA: 2024; EMA: NA; NMPA: NA
		2	NCT05060016	Active, not recruiting	SCLC	220	10 mg: CR: 1%, median RFS: 4.9 m, median OS: 14.3 m; 100 mg: CR: 8%, median RFS: 3.9 m, median OS: NR	
Zenocutuzumab	Merus	1/2	NCT02912949	Active, not recruiting	NRG1+ cancer	158	CR: 0.6%; median RFS: 6.8 m; median OS: NR	FDA: 2024; EMA: NA; NMPA: NA
		1/2	NCT04100694	Approved for marketing	NRG1+ PDAC	1	NA	
Catumaxomab	Fresenius Biotech and Trion Pharma	2/3	NCT00836654	Completed	MA	160	NA	FDA: NA; EMA: 2025; NMPA: NA
		2	NCT01504256	Completed	PC	42	NA	

RFS, relapse-free survival; NA, not available; NR, not reported; LBCL, large B cell lymphoma; MA, malignant ascites; CC, cervical cancer; GEJC, gastroesophageal junction cancer; GC, gastric cancer; PC, peritoneal carcinomatosis; NMPA, National Medical Products Administration.

### Clinical applications in hematological malignancies

#### Blinatumomab

Blinatumomab (Blincyto) is a BiTE that targets CD3 on T cells and CD19 on B cells. By bridging these cells, it activates T cells and induces tumor cell lysis without the need for costimulatory signals, while also promoting the proliferation and activity of CD8^+^ T effector memory cells.^73^

Blinatumomab has demonstrated robust and consistent efficacy across a range of clinical trials. In the study NCT01209286, a phase 2 trial in patients with relapsed or refractory (R/R) B-cell precursor (BCP)-acute lymphoblastic leukemia (ALL), 70% achieved complete response (CR)/CR with partial hematologic recovery (CRh), with 88% of responders attaining minimal residual disease (MRD) negativity and a median overall survival (OS) of 9.8 months.^87^ Similarly, NCT01466179 reported a CR/CRh rate of 43% in adults with Philadelphia chromosome (Ph)−ALL,^47^ while NCT01471782 yielded a 39% CR rate in pediatric R/R ALL.^88^ The pivotal phase 3 TOWER trial (NCT02013167) established the superiority of blinatumomab over chemotherapy, with a median OS of 7.7 months vs. 4.0 months (hazard ratio [HR], 0.71) and a higher CR rate (34% vs. 16%).^89^ In MRD-positive patients, the BLAST study (NCT01207388) demonstrated a 78% complete MRD response rate and a median OS of 36.5 months.^90^ In frontline settings, NCT02744768 revealed a 98% CR rate in newly diagnosed Ph+ ALL^91^; NCT02101853 reported 2-year disease free survival (DFS) and OS rates of 54.4% and 71.3%, respectively, with blinatumomab consolidation^92^; and NCT02003222 showed a 3-year OS of 85% when blinatumomab was combined with chemotherapy.^93^ Further supporting its broad utility, studies such as NCT03263572^94^ (with ponatinib), NCT01741792^95^ (in diffuse large B-cell lymphoma [DLBCL]), and NCT02000427^96^ also underscored its efficacy across hematologic malignancies, with ongoing evaluation in multiple myeloma (MM) (NCT03173430).

Based on compelling clinical trial data, blinatumomab received Breakthrough Therapy designation from the Food and Drug Administration (FDA) in July 2014, was granted priority review in October 2014, and received accelerated approval for R/R ALL on December 3, 2014. Subsequently, in March 2018, the FDA recognized MRD as an acceptable regulatory endpoint for B-cell malignancies in both adults and children, supported by results from the phase 2 BLAST trial.

#### Mosunetuzumab

Mosunetuzumab is a humanized BsAb with an aglycosylated non-functional Fc domain targeting CD20 and CD3, developed by Roche using KiH technology. This Fc region contains an amino acid substitution that prevents ADCC activation, potentially reducing the risk of cytotoxicity from therapy.^76^

Mosunetuzumab demonstrated long-lasting effects and a manageable safety profile in a substantial phase 2 study (NCT02500407) for patients with R/R B-cell non-Hodgkin lymphomas (B-NHLs) who had previously received at least two therapies.^97^ An article detailing the results of a phase 2 research assessing the safety and effectiveness of mosunetuzumab in 90 patients with R/R follicular lymphoma—all of whom had received two previous treatments—was published in The Lancet Oncology by Lihua Budde and colleagues. After a median follow-up duration of 18.3 months, mosunetuzumab alone resulted in an ORR of nearly 80%, with a remarkable 60% of patients achieving CR.^98^ In the study GO40516 (NCT03677141), mosunetuzumab was combined with polatuzumab vedotin, an anti-CD79b antibody-drug conjugate, for heavily pretreated patients with aggressive R/R B-NHL. This combination achieved an ORR of 65% and a CR rate of 48.3%.^99^ In phase 1b/2 trial (NCT03671018), Mosunetuzumab plus polatuzumab vedotin is an excellent second-line treatment option for patients with R/R large B cell lymphoma who are not candidates for a transplant due to its acceptable safety profile and highly lasting responses.^100^ Further clinical trials, such as NCT05169658 and NCT04246086, are exploring additional combination therapies. In June 2022, the EMA (European Medicines Agency) granted conditional marketing authorization for mosunetuzumab, followed by the FDA’s accelerated approval in December 2022.

#### Teclistamab

This IgG4-PAA BsAbs, known as teclistamab, targets both CD3 on T cells and BCMA on myeloma cells. It consists of anti-BCMA and anti-CD3 arms linked by disulfide bonds, enabling T cell-mediated cytotoxicity. Marketed as Tecvayli, it is used to treat relapsed or refractory MM (RRMM).^77^

The teclistamab-cqyv was tested in the multi-center, open-label, single-arm trial known as MajesTEC-1 (NCT03145181; NCT04557098). In this, 110 people who had already been through three treatments—including a proteasome inhibitor, an immunomodulatory drug, and an anti-CD38 monoclonal antibody—but who had never had BCMA-targeted therapy before were considered. With a median of five prior therapy lines, 77.6% of the 165 patients treated had triple-class refractory illness. The trial demonstrated an ORR of 63.0% with a CR or better achieved by 39.4% of patients at a median follow-up of 14.1 months. Additionally, 26.7% of patients showed MRD negativity, with an MRD negativity rate of 46% in those with CR or better. A median of 18.4 months was the duration of response (DOR), while 11.3 months was the median of progression-free survival (PFS).^74^^,^^101^ On August 24, 2022, the European Commission (EU) granted conditional marketing permission to teclistamab, making it the first first-in-class BsAb for MM to get approval on a global scale. On October 25, 2022, the FDA expedited the approval process.^102^ Ongoing studies are investigating the combination of teclistamab with other therapeutic agents to further explore its efficacy and potential in treating MM.

#### Glofitamab

Glofitamab, developed by Roche using CrossMab technology, features a unique 2:1 format with one CD3-binding scFv and two CD20-binding Fab regions, enhancing CD20 affinity. Its Fc region is engineered to minimize FcγR and C1q binding, reducing toxicity and prolonging half-life.^103^

In a phase 1 trial (NCT03075696) of patients with R/R B-NHLs, glofitamab monotherapy achieved an ORR of 70.5% and a CR of 47.7% in the follicular lymphoma subgroup.^48^ For R/R DLBCL patients in a phase 1/2 study, 39% achieved CR with a median follow-up of 12.6 months.^104^ The phase 3 STARGLO trial (NCT04408638) demonstrated that glofitamab combined with gemcitabine-oxaliplatin significantly improved OS compared to R-GemOx (median OS, 25.5 vs. 12.9 months; HR, 0.62).^105^ Based on these results, glofitamab received accelerated FDA approval in June 2023 and EU approval in July 2023 for R/R DLBCL after two or more prior systemic therapies.

#### Epcoritamab

Epcoritamab (GEN3013), developed by Genmab using DuoBody technology, is a subcutaneous-administered IgG-like BsAb targeting CD3 and CD20. Its Fc region contains silenced effector functions to enhance safety.^106^

In the pivotal EPCORE NHL-1 trial (NCT03625037) involving patients with R/R B-NHLs after at least two prior therapies, epcoritamab demonstrated an objective response rate (ORR) of 63% and a CR rate of 39% in the DLBCL cohort (*n* = 157).^107^^,^^108^ Based on these results, the FDA granted accelerated approval for R/R DLBCL in May 2023, followed by EMA approval in September 2023. The FDA further expanded its indication in June 2024 to include R/R follicular lymphoma after two or more systemic therapies, supported by data showing an ORR of 82% and CR of 63% in 128 follicular lymphoma (FL) patients.^109^ Ongoing studies continue to explore epcoritamab’s potential in combination regimens and expanded applications.

#### Talquetamab

Talquetamab, developed by Janssen using DuoBody technology, is a bispecific IgG antibody targeting CD3 and GPRC5D—a receptor overexpressed on plasma cells in MM. It represents the first T cell engager directed against GPRC5D for MM treatment.^110^

In the phase 1/2 MonumentT-1 trial (NCT03399799, NCT04634552), talquetamab demonstrated robust efficacy in heavily pretreated RRMM. Phase 2 results showed an objective response rate of approximately 60%–70% in patients who had received prior therapies including proteasome inhibitors, immunomodulatory drugs, and anti-CD38 antibodies.^111^ Based on these findings, the FDA granted accelerated approval to talquetamab in August 2023 for adult patients with RRMM who have undergone at least four prior lines of therapy. The European Union has also approved its use in patients who have received at least three prior regimens, including an immunomodulatory agent, a proteasome inhibitor, and an anti-CD38 antibody.

#### Elranatamab

A BsAb called elranatamab (Elrexfio) was created by Pfizer. It binds to BCMA on myeloma cells and CD3 on T cells, enhancing the ability of T cells to cytotoxically destroy cancer plasma cells.^112^

In the ongoing phase I MagnetisMM-1 human trial (NCT03269136), elranatamab demonstrated sustained clinical responses in patients with RRMM.^113^ The MagnetisMM-3 trial was a phase 2 multicenter, open-label, single-arm research (NCT04649359) that included 123 patients with RRMM who had already had treatment with an immunomodulatory medication, an anti-CD38 antibody, and at least one proteasome inhibitor. While 35% of patients achieved CR or better, the research indicated an ORR of 61%.^114^ Based on the positive outcomes from pivotal clinical trials, elranatamab received its first approval in the United States for the treatment of adult patients with RRMM.^115^ In addition, the drug was granted a positive opinion in the EU for the treatment of RRMS and is being reviewed in Japan and several other countries around the world.

### Clinical applications in solid tumors

#### Amivantamab (Rybrevant)

Amivantamab (Rybrevant) is a BsAb targeting both EGFR and MET receptors, developed for NSCLC with EGFR exon 20 insertion mutations. Its afucosylated Fc region enhances ADCC, engaging macrophages and NK cells while inhibiting receptor signaling.^59^

Amivantamab received FDA approval in May 2021^116^ based on the CHRYSALIS trial (NCT02609776), which evaluated the combination of amivantamab and lazertinib in EGFR-mutant NSCLC patients who had progressed on third-generation TKI (tyrosine kinase inhibitor) monotherapy but were chemotherapy naive.^117^ The ORR was 36% in an exploratory cohort of 45 patients.^118^ The PAPILLON study (NCT04538664) demonstrated that amivantamab with chemotherapy significantly improved PFS in advanced NSCLC with EGFR exon 20 insertions (median PFS, 11.4 months vs. 6.7 months; HR, 0.40; *p* < 0.001).^119^ The MARIPOSA-2 trial (NCT04988295) showed that combining amivantamab with chemotherapy, with or without lazertinib, improved PFS alone in EGFR-mutant NSCLC patients who had progressed after osimertinib therapy.^120^ Subcutaneous amivantamab-lazertinib also demonstrated non-inferiority to intravenous administration in a phase 3 trial (NCT05388669), with a consistent safety profile, fewer infusion-related reactions, and improved survival.^121^ In the phase 3 study (NCT04487080), amivantamab-lazertinib was more effective than osimertinib in patients with EGFR-negative advanced NSCLC, with significantly longer PFS (23.7 months vs. 16.6 months; *p* < 0.001) and median DOR (25.8 months vs. 16.8 months). Amivantamab, carboplatin, and pemetrexed was approved by the FDA as a combination for advanced NSCLC patients with EGFR exon 19 deleterious or exon 21 L858R mutation after EGFR TKI failure driven by these results.^122^

#### Tebentafusp

Tebentafusp (Kimmtrak), developed by Immunocore, is a bispecific TCR therapy specifically designed for treating unresectable or metastatic uveal melanoma, an aggressive and rare eye cancer.^123^

Compared to the control group, patients with untreated metastatic uveal melanoma had a median OS of 21.7 months in the tebentafusp group (HR, 0.51; *p* < 0.0001), according to the phase 3 IMCgp100-202 trial (NCT03070392).^124^ In NCT02570308, a study in 127 patients with metastatic uveal melanoma that did not respond to treatment, 62% survived for a year and the median survival was 16.8 months, despite an ORR of only 5%.^125^ In the NCT02535078 phase 1b trial, tebentafusp combined with durvalumab and/or tremelimumab in 85 HLA-A∗02:01^+^ patients with metastatic cutaneous melanoma showed a 14% response rate, 41% tumor shrinkage, and a 1-year OS rate of 76%.^126^ These findings led to the drug’s January 2022 FDA approval for the treatment of HLA-A∗02:01+ patients with metastatic or inoperable uveal melanoma.^127^

#### Ivonescimab

Ivonescimab (AK112), developed by Akeso Biopharmaceuticals, is a novel BsAb targeting both PD-1 and VEGF, enabling simultaneous immune checkpoint blockade and anti-angiogenic activity. This dual mechanism enhances antitumor immunity while inhibiting tumor vascularization.^128^

In the HARMONi-5 trial (NCT04900363) involving immunotherapy-naive advanced NSCLC patients, ivonescimab achieved an ORR of 39.8% and a DCR of 86.1%, with efficacy correlating to PD-L1 expression levels: ORRs were 14.7% (tumor proportion score [TPS] <1%), 51.4% (TPS ≥1%), and 57.1% (TPS ≥50%).^128^ The phase 1a study (NCT04047290) in advanced solid tumors reported a confirmed ORR of 25.5% and DCR of 63.8%.^78^ The phase 3 HARMONi-A trial (NCT05184712) demonstrated significantly improved PFS with ivonescimab plus chemotherapy vs. placebo in EGFR-mutant NSCLC after TKI failure (median PFS, 7.1 vs. 4.8 months; HR, 0.46; *p* < 0.001), along with a higher ORR (50.6% vs. 35.4%, *p* = 0.006)^129^ Based on these results, ivonescimab received approval from China’s National Medical Products Administration in May 2024 for EGFR-mutated non-squamous NSCLC following TKI progression and was granted FDA Fast Track designation in September 2024.

#### Cadonilimab

Cadonilimab is a BsAb made available by Akeso Biopharma. The design aims to concurrently target PD-1 and CTLA-4 (cytotoxic T-lymphocyte-associated antigen 4), two essential immune checkpoints that regulate T cell activation and immune response.

In metastatic NSCLC (NCT04172454), it achieved a 10% response rate in immunotherapy-naive patients.^130^ The COMPASSION-03 trial (NCT03852251) showed ORRs of 32.3% in cervical cancer, 18.2% in esophageal cancer, and 16.7% in hepatocellular carcinoma (HCC) ^131^ A phase 1 study (NCT03261011) reported a 13.4% ORR and 12.9-month DOR with favorable safety.^132^ In advanced HCC (NCT04444167), cadonilimab yielded a 35.5% response rate and 8.6-month median PFS.^51^ Combination therapy with anlotinib in NSCLC (NCT04646330) and monotherapy in metastatic cervical cancer (NCT04868708) showed promising efficacy, with response rates reaching 66.7%–92.3%.^133^^,^^134^ The COMPASSION-16 trial (NCT04982237) demonstrated significantly improved PFS in cervical cancer (12.7 vs. 8.1 months), leading to its conditional approval in China in June 2022 for relapsed or metastatic cervical cancer.^49^ Additional studies in gastric/gastroesophageal junction cancer (COMPASSION-04) further support its clinical utility, establishing cadonilimab as a valuable therapeutic option in the immuno-oncology landscape.

#### Tarlatamab

The first medication from this new class of therapeutics to be tested clinically in patients with small-cell lung cancer (SCLC) is tarlatamab, a BiTE molecule with an extended half-life that targets delta-like ligand 3.^135^

Tarlatamab was studied in the NCT03319940 study for R/R SCLC, and the results showed a DCR of 51.4%, a median DOR of 12.3 months, and an ORR of 23.4%. In addition, survivorship was 13.2 months on average and PFS was 3.7 months. The results were 25.0% ORR, 11.2 months median duration of response (mDOR), and 17.5 months median overall survival (mOS), according to the most recent statistics.^135^ The NCT05060016 phase 2 trial in previously treated SCLC patients reported an ORR of 40% (10 mg) and 32% (100 mg), with 59% of responders maintaining responses for at least 6 months. Median PFS was 4.9 months (10 mg) and 3.9 months (100 mg), with 9-month OS rates of 68% and 66%, respectively. May 16, 2024, was the day when the FDA announced the accelerated approval of tarlatamab-dlle (Imdelltra) for the treatment of extensive-stage SCLC that has progressed after platinum-based chemotherapy.^36^

#### Zenocutuzumab

Zenocutuzumab (MCLA-128) is a bispecific IgG1 antibody targeting HER2 and HER3, designed to inhibit neuregulin-1 (NRG1) fusion-driven signaling via a “dock-and-block” mechanism. It is developed using Merus’s Biclonics platform.^136^

Interim results from the ongoing phase 1/2 eNRGy trial (NCT02912949) and an early access program (NCT04100694) have demonstrated its clinical activity in NRG1-positive cancers.^137^^,^^138^ As of July 31, 2023, in 33 evaluable patients with NRG1+ pancreatic cancer (PDAC), the objective response rate (ORR) was 42.4%, with 82% experiencing tumor shrinkage. Among 78 evaluable NRG1+ NSCLC patients, the ORR was 37.2% and the median DOR was 14.9 months.

The US FDA has granted zenocutuzumab Breakthrough Therapy designation for advanced NRG1+ PDAC, Fast Track status for NRG1-fusion solid tumors, and Orphan Drug designation for PDAC. Merus anticipates submitting a Biologics License Application for NRG1+ PDAC and NSCLC by early 2024.

#### Catumaxomab

Catumaxomab (Removab), developed by Fresenius Biotech and Trion Pharma, is a rat-mouse hybrid BsAb targeting CD3 and EpCAM. It mediates T cell recruitment and activates Fcγ receptor-expressing accessory cells and is indicated for malignant ascites in patients with EpCAM-positive metastatic cancers.^139^

In a phase 2/3 trial (NCT00836654), catumaxomab significantly delayed quality of life deterioration compared to paracentesis alone (median 47–49 days vs. 19–26 days; *p* < 0.01) in patients with malignant ascites.^140^ A subsequent phase 2 study (NCT01504256) in gastric cancer patients with peritoneal carcinomatosis compared catumaxomab plus 5-fluorouracil, leucovorin, oxaliplatin and docetaxel (FLOT) chemotherapy vs. FLOT alone. While macroscopic complete remission rates (27% vs. 19%) and survival outcomes showed no statistically significant differences, the combination demonstrated feasible tolerability and supported further investigation in multimodal approaches.

Catumaxomab received EMA approval in April 2009. However, it was voluntarily withdrawn from the US market in 2013 and the EU market in 2017 for commercial reasons.

## Perspective and conclusion

While BsAbs have demonstrated potent antitumor efficacy and immune activation in recent years, their clinical application faces significant safety challenges, particularly CRS.^141^ Clinical trials have reported grade 3–4 CRS events, highlighting its significance as a dose-limiting toxicity.^88^ CRS manifests as a systemic inflammatory response ranging from flu-like symptoms to life-threatening shock, accompanied by laboratory abnormalities including cytopenia, elevated liver enzymes, and hyperferritinemia. This condition results from excessive cytokine release (IL-6, IL-1, IFN-γ, TNF) by immune and bystander cells, potentially leading to multi-organ damage.

Current management strategies prioritize early intervention with corticosteroids and cytokine-directed therapies. Tocilizumab, an IL-6 receptor antagonist approved by the FDA in 2017 for CRS management, effectively controls systemic symptoms without substantially compromising T cell function, although its efficacy against neurotoxicity remains limited due to poor blood-brain barrier penetration.^142^^,^^143^ Additional agents under investigation for refractory cases include siltuximab (anti-IL-6), anakinra (IL-1 receptor antagonist), and TNF-α inhibitors.^144^^,^^145^ Novel approaches such as suicide gene systems in engineered T cells represent promising strategies for mitigating life-threatening CRS episodes. These evolving management protocols reflect ongoing efforts to optimize the risk-benefit profile of BsAb therapies.

Despite notable clinical success in hematologic malignancies, BsAbs have demonstrated limited efficacy in solid tumors, primarily due to the immunosuppressive TME, heterogeneous antigen expression, and poor T cell infiltration.^70^^,^^146^ To address these challenges, next-generation BsAbs are being engineered for enhanced tissue penetration, conditional activation via protease- or pH-sensitive mechanisms, and stromal modulation to promote vascular normalization and immune cell recruitment. Given the complexity of solid tumors, BsAbs will likely require rational combination strategies to achieve durable responses. Promising approaches include pairing BsAbs with immune checkpoint inhibitors (e.g., anti-PD-1, -LAG-3, -TIGIT) to reverse T cell exhaustion, combining them with cancer vaccines to amplify antigen-specific immunity and integrating them with radiotherapy to enhance antigen release and immune cell infiltration into the TME.^147^^,^^148^

In addition, BsAbs may play disease stage-specific roles. In early-line therapy, BsAbs combined with chemotherapy or immunotherapy have shown promise in deepening responses and potentially reducing relapse risk in frontline settings—evidence from B-cell malignancies supports such combination approaches achieving high response rates even in aggressive disease.^149^ In relapsed/refractory contexts, where immune dysfunction is prevalent, combining BsAbs with immunomodulatory agents (e.g., Immune-mediated inflammatory diseases [IMiDs]) has mechanistic and emerging clinical support and may help restore T cell function and overcome resistance.^20^ Moreover, the MRD setting—characterized by low tumor burden—has been proposed as a favorable context for BsAb-mediated immune clearance, with recent lymphoma studies indicating that MRD negativity after BsAb therapy correlates with improved outcomes, although prospective, MRD-guided interventional data remain limited.^150^

As oncology moves toward precision medicine, BsAbs are well-positioned to enable tumor-targeted delivery of cytokines or co-stimulatory signals, remodel the immunosuppressive TME, and enhance immune cell infiltration. Future efforts will focus on biomarker-driven patient selection, optimized dosing, and rational combinations to maximize efficacy while minimizing toxicity. Advances in protein engineering are expected to yield next-generation BsAbs with refined pharmacological properties.

In conclusion, the future of BsAbs appears bright but will require multi-dimensional innovation—balancing efficacy with safety, overcoming the hurdles of solid tumors, and tailoring strategies to disease stage. With continued integration of mechanistic insights, translational advances, and clinical innovation, BsAbs have the potential to transform cancer immunotherapy and deliver durable benefit across both hematologic and solid malignancies.

## Acknowledgments

This work was supported by the 10.13039/501100001809National Natural Science Foundation of China (82403929, 82503827, and 8250101942), the 10.13039/501100004731Natural Science Foundation of Zhejiang Province (LQ24H160007), and the 10.13039/501100002858China Postdoctoral Science Foundation (no. GZB20230642, 2025T180593, and 2024M763330).

## Author contributions

S.Z. and F.L. performed the selection of literature, drafted the manuscript, and prepared the figures. M.N. collected the related references and participated in discussion. K.W., T.L., and M.Y. designed the work. All authors read and approved the final manuscript.

## Declaration of interests

The authors declare no competing interests.
